# Regulation of PrP^C^ signaling and processing by dimerization

**DOI:** 10.3389/fcell.2014.00057

**Published:** 2014-10-09

**Authors:** Xavier Roucou

**Affiliations:** Department of Biochemistry, Faculty of Medicine, Université de SherbrookeSherbrooke, QC, Canada

**Keywords:** prion protein trafficking, dimerization, signaling, neuroprotection, neurodegeneration

## Abstract

The cellular prion protein (PrP^C^) is a glycosylphosphatidylinositol (GPI)-anchored protein present at the cell surface. PrP^C^ N-terminal moiety is intrinsically disordered and is able to interact with a variety of ligands. Physiological ligands have neurotrophic activity, whilst others, including protein toxic oligomers, have neurotoxic functions. These two opposite activities involve different interacting partners and result from different PrP^C^-activated signaling pathways. Remarkably, PrP^C^ may be inactivated either by physiological endoproteolysis and release of the N-terminal domain, or by ectodomain shedding. Ligand-induced PrP^C^ dimerization or enforced dimerization of PrP^C^ indicate that PrP^C^ dimerization represents an important molecular switch for both intracellular signaling and inactivation by the release of PrP^C^ N-terminal domain or shedding. In this review, we summarize evidence that cell surface receptor activity of PrP^C^ is finely regulated by dimerization.

## Introduction

PrP^C^ is a cell surface protein with a bipartite structure: the N-terminal domain is disordered and the C-terminal domain is structured and contains three α-helices and two short β-strands (Wuthrich and Riek, 2001). The physiological function of PrP^C^ is unclear, but a large body of evidence indicates that PrP^C^ is a neuroprotective and neurotrophic protein (Linden et al., 2008). The neuroprotective function of PrP^C^ against different insults was demonstrated *in vitro* in primary neurons and in cell lines, and *in vivo* (Roucou and LeBlanc, 2005; Lo et al., 2007). In these studies, PrP^C^ expression was able to slow or halt cell death whilst PrP^C^ absence did not prevent cell death. In a pioneer investigation, PrP^C^ expression prevented cell death triggered by serum deprivation of immortalized hippocampal neurons (Kuwahara et al., 1999). Subsequent studies provided significant evidence for the implication of PrP^C^ in cell survival. In addition to these neuroprotective effects, PrP^C^ regulates cell proliferation, differenciation, growth, and PrP^C^ is also important for the expansion of stem cells in culture (Martins et al., 2010; Miranda et al., 2013). Some of these trophic mechanisms have been addressed and involve the assembly of protein complexes at the cell surface.

Most of neuroprotective and neurotrophic activities result from PrP^C^-mediated signaling (Martins et al., 2010; Schneider et al., 2011). Thus, a large body of data indicate that GPI-anchored PrP^C^ is a cell surface receptor or co-receptor and that its engagement with one of its numerous ligands or with antibodies activates different intracellular pathways. Cell surface receptors are generally activated by dimerization (Heldin, 1995), and this may also be valid for PrP^C^ which forms dimers in native conditions and can be experimentally engaged with cross-linking antibodies (Mouillet-Richard et al., 2000; Rambold et al., 2008).

In prion diseases, PrP^C^ changes conformation into a pathological conformer termed PrP^Sc^ (Prusiner, 1998). The exact mechanism of this conformational change or prion conversion is unclear but may involve the initial formation of dimers. During the process of prion conversion, PrP^Sc^ oligomerizes and form toxic oligomers that interact with PrP^C^ and switch its neuroprotective/neurotrophic signaling to a neurotoxic signaling (Rambold et al., 2008; Resenberger et al., 2011).

In this review, I will summarize some of the most important studies on the role of dimerization on the physiological and pathological function of PrP^C^ and PrP^Sc^, respectively.

## PrP^C^ dimerization and cell signaling (Figure 1)

### Detection of PrP^C^ dimers in native conditions and cytoprotection

PrP^C^ dimers were detected in solution in a partially purified fraction from normal bovine brain thalamus (Meyer et al., 2000), and in murine neuroblastoma N2a cells expressing Syrian hamster PrP^C^ (Priola et al., 1995). Syrian hamster, human and bovine PrP^C^ expressed in baculovirus and purified under native conditions spontaneously form dimers (Hundt et al., 2003). Dimerization of human PrP^C^ was confirmed in BHK cells overexpressing PrP^C^ and in yeast two-hybrid assays (Hundt et al., 2003). More recently, endogenous PrP^C^ dimers were also detected by blue native PAGE in N2a cells and the dimerization domain mapped to a hydrophobic domain of the protein (amino acids 112-MAGAAAAGAVVGGLGGYMLGSA-133) (Rambold et al., 2008). Finally, PrP^C^ dimers were detected after chemical crosslinking in crude membranes from human neuroblastoma SH-SY5Y cells and mouse brains (Rambold et al., 2008). These results convincingly demonstrate that PrP^C^ has an intrinsic tendency to dimerize in native conditions and suggest that dimerization is important for the physiological function of PrP^C^.

The assembly of natural PrP^C^ dimers at the plasma membrane is associated with protective activity against the excitotoxin kainate and altering dimer formation results in cell death (Rambold et al., 2008). Based on their own data and previous data showing PrP^C^-mediated signaling using anti-PrP^C^ antibodies (see below), the authors proposed that cell surface PrP^C^ dimers induce protective signaling through an unknown transmembrane receptor. This study did not elucidate whether the formation of dimers is constitutive or depends on an unknown ligand. Also, the proportion of PrP^C^ dimers is unknown.

### Antibody-induced PrP^C^ dimerization reveals the signaling pathway controlled by PrP^C^

Antibody-induced dimerization (also termed antibody-induced ligation or -cross-linking) is used to mimic an extracellular signal on cell surface receptors and trigger signal transduction. Although it is unclear if such strategy mimics the interaction with a partner or dimerization of the receptor, antibody-induced dimerization is largely used to engage a receptor in the absence of its ligand and relays intracellular signals. GPI-anchored proteins associate with raft domains in the plasma membrane and activate signal transduction pathways upon engagement with ligands or via antibody-induced dimerization (Robinson, 1991; Suzuki et al., 2012). For GPI-anchored proteins, signal transduction occurs through activation of intracellular tyrosine kinases including the Src-family kinases (Stefanova et al., 1991; Chen et al., 2006). Mouillet-Richard et al were the first to show that engagement of PrP^C^ using an antibody-induced dimerization approach activates a Fyn-dependent signaling pathway in serotonergic and noradrenergic mouse cells differenciated from the murine neuroectodermal progenitor 1C11 clone (Mouillet-Richard et al., 2000). Similar results were obtained with two different antibodies, 1A8 and SAF61 targeting C-terminal epitopes. Using 4 different antibodies to induce PrP^C^ dimerization, SAF61, Bar221, and 1A8 that target C-terminal epitopes, and SAF32 which targets epitope 79-92, NADPH oxidase was subsequently identified as the main primary target of PrP^C^-mediated signaling. NADPH oxidase-dependent reactive oxygen species production stimulated the phosphorylation of extracellular regulated kinases 1/2 (Erk1/2) in the 1C11 neuroectodermal precursor and its neuronal differentiated progenies, the hypothalamic GT1-7 cells, and the T lymphoid BW5147 cells (Schneider et al., 2003). PrP^C^ signaling was dependent on Fyn in neuronal cells only, indicating specificity in the control of PrP^C^ function. PrP^C^-mediated phosphorylation of Erk1/2 was independently confirmed in GT1-7 neuronal cells (Monnet et al., 2004) and in human neuroblastoma SH-SY5Y cells (Rambold et al., 2008). PrP^C^-induced ROS production and Erk1/2 phosphorylation was confirmed using an inducible dimerization strategy (Beland et al., 2012).

These studies lend support for a role of PrP^C^ in signal transduction and further investigations provided more insight into the physiological consequence of PrP^C^ signaling in neuronal 1C11 cells. In 1C11 serotonergic cells expressing 5-HT_2B_, 5-HT_1B/D_, and 5-HT_2A_ receptor subtypes. PrP^C^ dimerization interfered with the signaling activity of these three serotonergic receptors belonging to the GPCR family likely by modulating the recruitment of G-proteins (Mouillet-Richard et al., 2005). PrP^C^ dimerization promoted the recruitment of the cAMP responsive element binding protein (CREB) transcription factor and the transcription of several genes with important function in cellular protection and neuronal plasticity (Pradines et al., 2008). In addition, PrP^C^ dimerization inactivated the Glycogen Synthase Kinase 3β and activated serotonergic signaling through inhibition of the serotonin 1B receptor (Hernandez-Rapp et al., 2014). CREB recruitment and GSK3β are generally associated with cytoprotection, suggesting an important function of PrP^C^ in cell survival and homeostasis.

For several years, these data were in contradiction with previous results indicating that antibody-induced PrP^C^ dimerization is neurotoxic *in vivo* (Solforosi et al., 2004). However, these results were later invalidated with similar and other antibodies (Klohn et al., 2012). This debate is still ongoing since a recent study demonstrated that anti-PrP^C^ antibodies induce rapid neurotoxicity in mice and cerebellar organotypic cultured slices (Sonati et al., 2012). Importantly, PrP^C^ dimerization is unlikely to be involved in neuronal toxicity since single-chain antibodies were also toxic.

### PrP^C^ signaling activated by different ligands

At the cell surface, PrP^C^ interacts directly or indirectly with a variety of ligands as diverse as metals, lipids, nucleic acids, glycosaminoglycans, and other proteins (Linden et al., 2008; Beland and Roucou, 2012). In physiological conditions, it was proposed that PrP^C^ is a scaffolding protein providing essential molecular interactions and signaling neurotrophic activities (Martins et al., 2010). PrP^C^ ligands promoting neurotrophic activity include laminin, the 37-kDa/67-kDa laminin receptor precursor/laminin receptor, vitronectin, the neural cell adhesion molecule, and the Stress Inducible Protein 1 (Martins et al., 2010).

In pathological conditions, binding of PrP^Sc^ to cell surface PrP^C^ corrupts PrP^C^ signaling and results in cellular toxicity (Rambold et al., 2008; Resenberger et al., 2011). This finding is particularly important as it provides a simple explanation for the observation that PrP^C^ on the cell surface is critical for the neurotoxicity of PrP^Sc^ in prion diseases (Brandner et al., 1996; Chesebro et al., 2005). PrP^C^ dimerization is essential for the toxicity of PrP^Sc^ (Rambold et al., 2008). PrP^C^ is also a receptor for other toxic β-sheet oligomers, including Aβ in Alzheimer's disease (Lauren et al., 2009; Gunther and Strittmatter, 2010; Resenberger et al., 2011).

## PrP^C^ integrity at the cell surface is regulated by proteolysis and dimerization: PrP^C^ metabolites and neuroprotection (Figure 1)

### PrP^C^ is a target for several posttranslational endoproteolytic events

Following translocation into the endoplasmic reticulum, signal peptidase removes a N-terminal signal peptide, and a C-terminal peptide is removed prior to the attachment of the GPI anchor. Thus, human PrP^C^ is translated as an immature 253 amino acids protein and mature PrP^C^ is a 208 residues protein. After trafficking through the secretory pathway, a fraction of PrP^C^ may undergo three proteolytic cleavages (Altmeppen et al., 2012). An α-cleavage between residues 110–111 and 112 in a late compartment of the secretory pathway produces PrPC1, a 17 kDa GPI-anchored C-terminal polypeptide, and a 11 kDa N-terminal polypeptide released in the extracellular space. The identity of the protease responsible for α-cleavage, termed the α-PrPase (Oliveira-Martins et al., 2010), is still unclear. A β-cleavage at amino acids 89/90 generates PrPC2, a 20 kDa GPI-anchored polypeptide, and the corresponding 8 kDa PrPN2 fragment. β-cleavage occurs at the cell surface mainly in pathological conditions; calpains execute β-cleavage in prion diseases whilst reactive oxygen species perform β-cleavage under conditions of oxidative stress. In addition, a fraction of PrP^C^ is constitutively shed from the cell surface after proteolytic cleavage close to the GPI anchor. *In vivo*, the main protease responsible for PrP^C^ shedding is the zinc metalloproteinase ADAM10 (Altmeppen et al., 2012).

### Neuroprotective PrP^C^-derived PrPN1 and PrPC1 metabolites

In recent years, α-cleavage attracted a lot of attention because it results in the production of PrPN1, a natural PrP^C^ metabolite with a clear neuroprotective activity against different insults *in vivo*, in primary neuronal cultures and in cell lines (Guillot-Sestier et al., 2009, 2012; Resenberger et al., 2011; Beland et al., 2012; Fluharty et al., 2013; Beland and Roucou, 2013a). In particular, the neuroprotection against soluble Aβ oligomers that may be the culprit species in Alzheimer's disease may pave the way for the discovery of a new class of therapeutic molecules (Beland et al., 2012; Fluharty et al., 2013). There is also some evidence that α-cleavage is increased in post-mortem brain tissues of Alzheimer's disease patients, and that PrPN1 traps Aβ into amorphous aggregates unable to transform into soluble and toxic Aβ oligomers, and that α-cleavage decrease promotes neurotoxicity in prion and Alzheimer's diseases (Pietri et al., 2013; Beland et al., 2014). PrPN1 also binds to and antagonizes the toxicity of other β-sheet rich oligomers, including PrP^Sc^ oligomers, and PrPN1-derived therapeutic molecules may help treat different neurodegenerative disorders (Resenberger et al., 2011).

The GPI-anchored PrPC1 fragment after α-cleavage of PrP^C^ protects against prion infection of neuronal and non-neuronal cell lines and acts as a dominant-negative inhibitor of prion conversion *in vivo* (Lewis et al., 2009; Westergard et al., 2011). The mechanism of action of PrPC1 is unclear, but since PrPC1 is resistant to prion conversion, the authors proposed that PrPC1 competes with PrP^C^ for binding to infectious PrP^Sc^ (Westergard et al., 2011). Thus, PrPC1-derived peptides may have therapeutic benefits in prion diseases.

### PrP^C^ dimerization stimulates its trafficking to the plasma membrane and the production of PrPN1 and PrPC1

As many experimental data converge to support the proposition that PrPN1 and PrPC1 are neuroprotective metabolites, two therapeutic avenues could be proposed in prion diseases: to provide exogenous PrPN1- or PrPC1-derived molecules, or to increase the natural production of PrPN1 and PrPC1 by stimulating the α-cleavage. The α-cleavage mechanism is nebulous and only two elements are known: it occurs in the late secretory pathway but the enzyme is still unknown, and the hydrophobic domain is essential for this cleavage (Bremer et al., 2010; Oliveira-Martins et al., 2010). This domain is also essential for the physiological dimerization of PrP^C^ (Rambold et al., 2008), supporting the hypothesis of a possible connection between dimerization and α-cleavage. Using an inducible dimerization strategy with a permeable dimerizer, we were able to show that PrP^C^ dimerization in cell lines and primary neurons increase PrP^C^ trafficking to the plasma membrane and largely increase the production of PrPN1, PrPC1, and shed PrP^C^ (Beland et al., 2012). After dimerization, conditioned medium containing these three metabolites strongly protected cells against toxic Aβ oligomers.

Since levels of the products of both α-cleavage and shedding rose after dimerization, we concluded that the large increase of PrP^C^ trafficking to the plasma membrane was sufficient to explain the high levels of its metabolites. This effect was fast and occurred 4 h post-dimerization. Deletion of the hydrophobic domain, the natural dimerization domain, does not prevent PrP^C^ trafficking to the plasma membrane (Winklhofer et al., 2003). Thus, dimerization is not essential for PrP^C^ trafficking. We proposed a model with a constitutive and dimerization-independent pathway for PrP^C^ secretion, and a pathway regulated by dimerization. This regulated pathway would allow the cells to quickly respond to toxic insults by increasing the levels of protective PrP^C^ metabolites (Beland and Roucou, 2013a,b).

## The dark side of PrP^C^ dimerization revealed from *in vitro* prion conversion assays

PrP^C^→PrP^Sc^ conversion or prion conversion is central to prion diseases; this process is neurotoxic and PrP^Sc^ molecules assemble into infectious particles responsible for the transmission of the disease (Prusiner, 1998; Mallucci et al., 2003). Not surprisingly, numerous mechanistic studies have addressed this process using recombinant PrP (recPrP) and several experimental data have indicated an intrinsic tendency of the protein to form dimers during the initial steps of prion conversion. A fraction of Syrian hamster recPrP(90-231) forms alpha-helical dimers in solution in the presence of submicellar concentrations of SDS (Jansen et al., 2001). These dimers believed to be intermediates in prion conversion were observed by size exclusion chromatography, chemical crosslinking and analytical ultracentrifugation (Kaimann et al., 2008; Stöhr et al., 2008; Jansen et al., 2001). In these studies, non-denaturing concentrations of SDS were used to mimic membrane-like features. Using a reduction-oxidation protocol to induce the fibrillar assembly of Syrian hamster PrP(90–231), Lee and Eisenberg also observed the presence of dimeric intermediates in polyacrylamide native gels (Lee and Eisenberg, 2003). A similar conclusion was obtained with murine recPrP(23–231). During the conversion of murine PrP(23–231), an intermediate water-soluble β-sheet isoform termed PrP^β^ was identified (Luhrs et al., 2006). The kinetics of PrP^C^→PrP^β^ conversion suggest that dimerization is the rate-limiting step for the transition. The dimerization of murine recPrP(23–231) as a key molecular step during the conversion was confirmed in a subsequent study (Hafner-Bratkovic et al., 2011). Additionally, 3D reconstruction of murine recPrP(91–230) amyloid fibrils led to the proposition that dimers represent building units of such fibrils (Tattum et al., 2006).

These experiments were performed with non-posttranslationally modified PrP^C^. Yet *in vivo*, PrP^C^ carries two N-glycosylations and a GPI anchor and these complex posttranslational modifications may play an important role in prion conversion. To address this issue, posttranslationally modified PrP^C^ was purified from Chinese hamster ovary cells overexpressing Syrian hamster PrP^C^. This native PrP^C^ spontaneously formed dimers stabilized by intermolecular β-sheets after insertion into artificial membranes (Elfrink et al., 2008).

Altogether, these studies support the hypothesis that dimerization is an important step for prion conversion but they did not directly test this hypothesis. To address this issue, two strategies have been used. First, two monomeric mouse PrP (23–231) were covalently linked with a linker and recombinantly purified. This tandem protein oligomerized after purification, and thioflavine T staining indicated that such oligomers were likely on the pathway of amyloid formation, although this was not demonstrated (Simoneau et al., 2007). In a second strategy, we used a conditional dimerization approach to induce chemical dimerization of mouse recPrP(23–231) and human recPrP (23-231). α-helical PrP dimers spontaneously converted into β-sheet oligomers and amyloid fibrils were detected by electron microscopy and thioflavinT staining (Roostaee et al., 2009). Importantly, these experiments were performed in physiological-like conditions in the absence of any detergent or chaotropic agents.

Models of PrP dimers and the role of dimers in prion conversion are available (Warwicker, 2000; Gauczynski et al., 2001; Tompa et al., 2002). However, in all the above studies, prion conversion was assessed by the formation of PrP amyloid fibrils and/or partial resistance to proteinase K rather than by the formation of infectious PrP^Sc^ in animal bioassays. Hence, the biological significance of PrP^C^ dimerization for the formation of infectious PrP^Sc^ remains speculative.

## Conclusion

**Figure 1:**
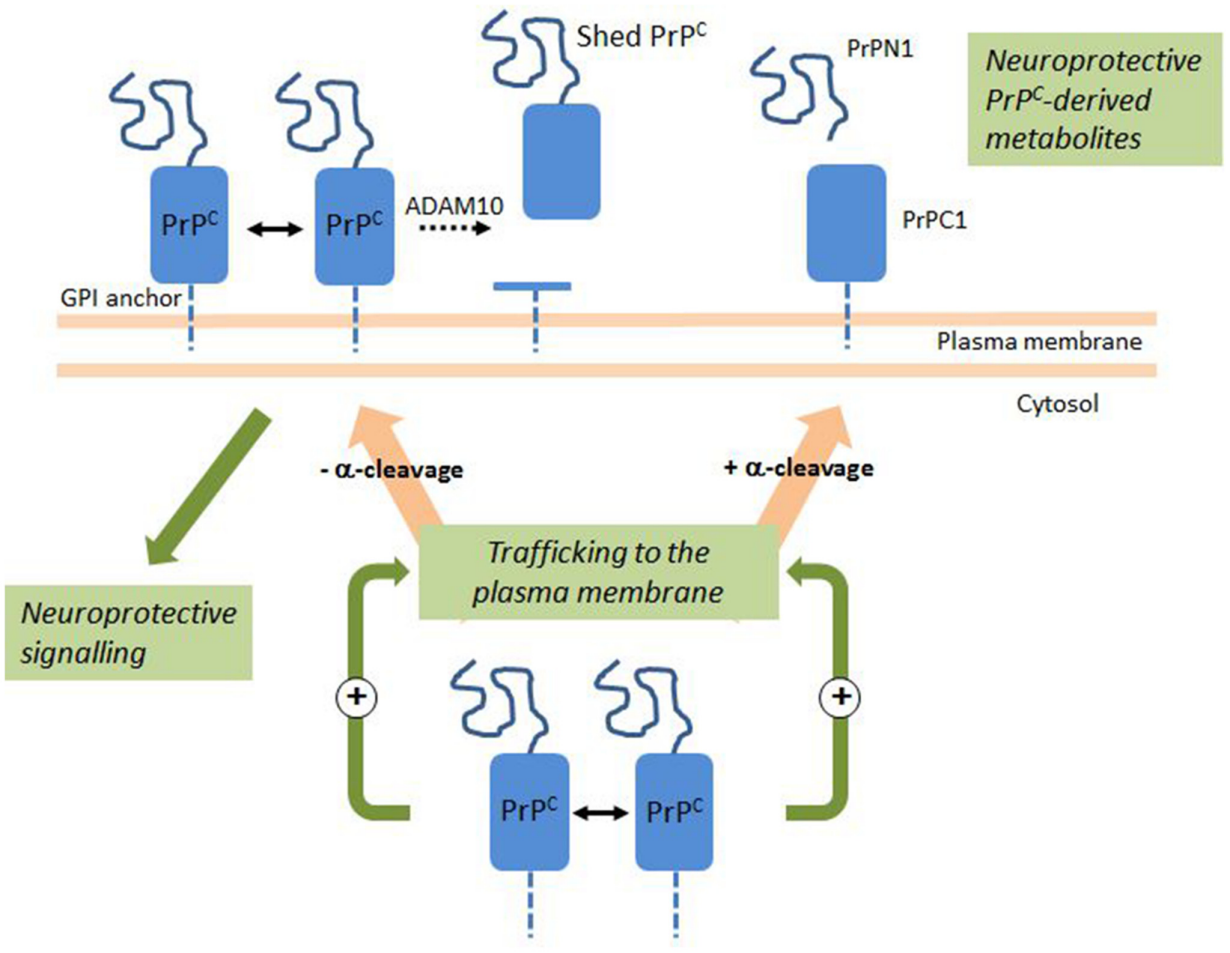
**PrP^C^ Dimerization activates a neuroprotective signaling pathway and the production of neuroprotective PrP^C^-derived metabolites**. En route to the plasma membrane, a fraction of PrP^C^ undergoes a physiological cleavage termed α-cleavage. Intracellular PrP^C^ dimerization stimulates trafficking to the plasma membrane and the release of PrPN1 and PrPC1 at the cell surface. PrP^C^ dimerization at the cell surface activates an intracellular signaling pathway with a neuroprotective outcome. At the cell surface, a fraction of PrP^C^ undergoes ADAM10-mediated shedding. Double arrows indicate dimerization; dotted arrow indicates shedding. See text for details.

PrP^C^ forms dimers in native conditions and cell surface dimerization clearly regulates PrP^C^-mediated signaling and the resulting physiological neuroprotective/neurotrophic activities (Rambold et al., 2008). Intracellular dimerization also drastically increases its trafficking to the plasma membrane and the production of its natural metabolites PrPN1 and PrPC1 (Beland et al., 2012). The combination of these two effects of PrP^C^ dimerization likely provides PrP^C^ with a powerful neuroprotective/neurotrophic function (Figure 1). However, the flip side of the coin is that unwanted dimerization may initiate prion conversion and result in neuronal toxicity (Tompa et al., 2002). Regulating PrP^C^ dimerization may help translate these findings into novel therapeutic interventions in neurodegenerative diseases (Beland and Roucou, 2013b).

### Conflict of interest statement

The author declares that the research was conducted in the absence of any commercial or financial relationships that could be construed as a potential conflict of interest.
